# Effectiveness of a Multicomponent Program for Fibromyalgia Patients in a Primary Care Setting (FIBROCARE Program): A Pragmatic Randomized Controlled Trial

**DOI:** 10.3390/jcm14010161

**Published:** 2024-12-30

**Authors:** Rosa Caballol Angelats, Alessandra Queiroga Gonçalves, Rosa Abellana, Noèlia Carrasco-Querol, Anna Pàmies Corts, Gemma González Serra, Dolors Gràcia Benaiges, Maria Cinta Sancho Sol, Immaculada Fusté Anguera, Susana Chavarria Jordana, Blanca Cuevas Baticón, Gemma Batlle Escolies, Maria Fibla Reverté, Noemí Espuny Vallés, Núria Buera Pitarque, Montserrat Martí Cavallé, Nuria Piñana Suazo, Joan Estivill Bargalló, Maria Àngels López Guerrero, Carolina López Guerrero, Pilar Pérez Acín, Immaculada Matamoros Callarisa, Jordi Baucells, Adrià Suazo Ciurana, José Fernández-Sáez, M. Rosa Dalmau Llorca, Anna Berenguera, Carina Aguilar Martín

**Affiliations:** 1 Unitat d’Expertesa en Síndromes de Sensibilització Central Terres de l’Ebre, Institut Català de la Salut, 43500 Tortosa, Spain; rcaballol.ebre.ics@gencat.cat (R.C.A.);; 2 Programa de Doctorat Medicina i Recerca Translacional, Departament de Fonaments Clínics, Facultat de Medicina, Universitat de Barcelona, 08036 Barcelona, Spain; 3 Direcció d’Atenció Primària Terres de l’Ebre, Institut Català de la Salut, 43500 Tortosa, Spain; 4 Unitat de Suport a la Recerca Terres de l’Ebre, Fundació Institut Universitari per a la Recerca al’Atenció Primària de Salut Jordi Gol i Gurina (IDIAPJGol), 43500 Tortosa, Spain; 5 Departament de Fonaments Clínics, Facultat de Medicina, Universitat de Barcelona, 08036 Barcelona, Spain; 6 Servei de Reumatologia, Hospital de Tortosa Verge de la Cinta, 43500 Tortosa, Spain; 7 Servei de Rehabilitació i Medicina Física, Hospital de Tortosa Verge de la Cinta, Gerència Territorial de Terres de l’Ebre, Institut Català de la Salut (ICS), 43500 Tortosa, Spain; ggonzalezs.ebre.ics@gencat.cat (G.G.S.);; 8 Centre de Salut Mental d’Adults, Fundació Pere Mata Terres de l’Ebre, 43500 Tortosa, Spain; 9 Equip d’Atenció Primària Tortosa Est, Institut Català de la Salut, 43500 Tortosa, Spain; 10 Equip d’Atenció Primària L’Ametlla de Mar–El Perelló, Institut Català de la Salut, 43860 l’Ametlla de Mar, Spain; 11 Equip d’Atenció Primària L’Aldea-Camarles-L’Ampolla, Institut Català de la Salut, 43896 L’Aldea, Spain; 12 Equip d’Atenció Primària Amposta, Institut Català de la Salut, 43870 Amposta, Spain; 13 Equip d’Atenció Primària Terra Alta, Institut Català de la Salut, 43780 Gandesa, Spain; 14 Equip d’Atenció Primària Móra la Nova-Móra d’Ebre, Institut Català de la Salut, 43770 Móra la Nova, Spain; 15 Equip d’Atenció Primària Flix, Institut Català de la Salut, 43750 Flix, Spain; 16 Equip d’Atenció Primària La Ràpita-Alcanar, Institut Català de la Salut, 43540 La Ràpita, Spain; 17 Direcció de Sistemes d’Informació i Comunicació, Gerència Territorial Terres de l’Ebre, Institut Català de la Salut, 43500 Tortosa, Spain; 18 Unitat Docent de Medicina de Familia i Comunitària Tortosa-Terres de l’Ebre, Institut Català de la Salut, 43500 Tortosa, Spain; 19 Campus Terres de l’Ebre, Universitat Rovira i Virgili (URV), 43500 Tortosa, Spain; 20 Equip d’Atenció Primària Tortosa Oest, Institut Català de la Salut, 43500 Tortosa, Spain; 21 Unitat Transversal de Recerca, Fundació Institut Universitari per a la Recerca al’Atenció Primària de Salut Jordi Gol i Gurina (IDIAPJGol), 08007 Barcelona, Spain; 22 Departament d’Infermeria, Universitat de Girona, 17003 Girona, Spain; 23 Unitat d’Avaluació, Direcció d’Atenció Primària Terres de l’Ebre, Institut Català de la Salut, 43500 Tortosa, , Spain

**Keywords:** fibromyalgia, outcome assessment, health care, rehabilitation, health-related quality of life, health services research

## Abstract

**Background/Objectives**: Multicomponent, non-pharmacological therapies are the preferred first-line treatments for fibromyalgia (FM), but little is known about them in primary care settings. Our study assessed the effectiveness of the FIBROCARE Program in improving the quality of life, functional impact, mood, and pain of people with FM. **Methods**: We conducted a pragmatic, randomized controlled trial that was not blinded for both patients and the professionals delivering the treatments in the study groups. We compared a group receiving non-pharmacological multicomponent group therapy (MT) based on health education, physical exercise, and cognitive–behavioral therapy with another group receiving the usual clinical care. The MT group was treated in the primary care context in Catalonia (Spain) through 12 consecutive weekly sessions. Both groups were followed up with at the end of the MT group sessions and 6 and 12 months after the group sessions with the Short-Form 36 (SF-36) v2 Health Survey questionnaire, the Hospital Anxiety and Depression Scale (HADS-A and HADS-D), the Visual Analog Scale, and the Revised Fibromyalgia Impact Questionnaire (ClinicalTrials.gov: NCT04049006). **Results**: Improvements in pain intensity, functional impact, physical health, fatigue, and emotional problems that affect daily activities in the MT group lasted up to 12 months. Benefits measured by the SF-36 Mental Health dimension and the HADS-A subscale were lost after 6 months. Effects on the SF-36 Social Functioning dimension and HADS-D present at 6 months persisted for up to 12 months. Generally, the longer the time since the FM diagnosis, the better was a patient’s mood. **Conclusions**: The FIBROCARE Program effectively improves all the studied health outcomes except patient mood, since anxiety symptoms persist. The program should reinforce patient psychological support overall, focusing particularly on the years initially after diagnosis.

## 1. Introduction

Fibromyalgia (FM) is a syndrome of central sensitization, characterized by chronic pain, fatigue, and loss of function. It involves a physical limitation of the patient with consequent effects on their state of mind and quality of their sleep, and has repercussions for their social and work environments and their quality of life [1,2]. Worldwide FM prevalence is 2% to 3% [3] and predominantly affects women; in Spain, 4.49% of women compared with 0.29% of men are affected [4].

Several diagnostic criteria for FM have been proposed in recent years, with the 2016 American College of Rheumatology criteria [5] so far performing the best [6]. However, in practice, it is difficult to deal with FM due to factors such as the lack of a diagnostic test that can confirm the clinical symptoms, the uncertainty about its etiopathogenesis, the non-acceptance of the disease among the medical community as a whole, and the social stigmatization of patients [7,8].

Non-pharmacological therapies are considered the first-line treatment [9,10]. The evidence for the most appropriate non-pharmacological treatment for FM indicates that those based on multicomponent and multidisciplinary therapies combined with individual follow-up give the best results [11,12,13].

The key multicomponent therapies appear to be those that incorporate an education program and a comprehensive physical exercise program along with aerobic exercise, stretching, relaxation, strengthening, and resistance, in a way that involves the whole body. Another important component is cognitive–behavioral therapy (CBT) for self-management that includes occupational therapy, moderation, acceptance, commitment, and motivation for change [11,13]. However, few studies have been designed as randomized controlled trials (RCTs), and there is a lack of evidence about the duration of the effect of multicomponent therapies on health outcomes related to this chronic pathology [14].

The creation of the 18 Units Specialized in Central Sensitivity Syndromes (USCSSs) in 2016 in Catalonia (Spain) [15] provided an opportunity to implement new programs for FM patients. In our case, as a unit created as part of primary health care (PC), it constitutes an opportunity to promote quality care at this level of the public health system. For this reason, we have designed a new program for FM patients, the FIBROCARE Program, that was designed [16] to be evaluated by a mixed-method study comprising a pragmatic RCT and a subsequent qualitative assessment of patients and professionals [17,18,19]. The present study aims to evaluate, through the RCT, the effectiveness of the program, a non-pharmacological multicomponent group therapy (MT) for improving the quality of life, functional impact, mood, and pain of people with FM.

## 2. Materials and Methods

Below, we summarize the methodology of the pragmatic RCT (parallel group type) that aimed to compare the usual clinical care (control group) with the effect of the MT on those who received, in addition to the usual clinical care, health education, physical exercise, and CBT (ClinicalTrials.gov: NCT04049006). In this study, neither patients nor treatment providers were blinded to group assignment. The usual clinical care for FM patients consisted of a non-standardized individual approach, without reference to any standardized health route. It was largely based on pharmacological treatment (analgesics, antidepressants, medication to aid sleep, some muscle relaxants) but could also be accompanied by some recommendations, offered on an ad hoc basis, for adapted regular physical exercise and psychological support.

### 2.1. The FIBROCARE Program

The multicomponent program was designed by the USCSS professionals from the Terres de l’Ebre Territorial management team of the Institut Català de la Salut (ICS) in collaboration with professionals (nurses and general practitioners) working in PC. These staff were trained as FM experts to carry out the program in each of the 11 PC center (PCCs) teams under this management, and to act as advisers to other professionals in their teams. The training, consisting of an initial course followed by annual training, was the first step of the program. Once trained, the FM experts carried out the program with the support of the USCSS professionals through 12 consecutive weekly sessions of 2 h duration. Program sessions were conducted face-to-face in the PCCs. Details of the program and its sessions have been provided previously [16,19] and are provided in Supplemental material S1.

### 2.2. Study Population

The patients studied were attended to in one of the 11 PCCs of the ICS, Terres de l’Ebre Territorial region, and had been diagnosed with FM (International Classification of Diseases—10 codes: M79.0, M79.7). Inclusion and exclusion criteria were applied to select patients for inclusion in the program, as previously described [16].

### 2.3. Recruitment, Data Collection, and Study Variables

The RCT was conducted in units, each with a pair of study groups of 10 to 12 participants. At baseline, patients who met the criteria were invited to an initial interview in which the study was explained, and their informed consent was requested. On provision of the latter, patients were assigned to one of the study groups according to a randomization list and received orientation about the activities planned for their group. Randomization was performed in each unit as previously described [16]. A set of questionnaires was delivered to be filled out face-to-face at baseline, at the end of the MT group sessions (=3 months after the beginning of the study), and 6 and 12 months after the MT group sessions. The set comprised an ad hoc questionnaire eliciting baseline clinical and sociodemographic data, the Short-Form 36 (SF-36) v2 Health Survey questionnaire [20] (Optum, Inc.(Johnston, RI, USA); license number QM048943), the Revised Fibromyalgia Impact Questionnaire (FIQR) [21,22], the Visual Analog Scale (VAS) [23,24], and the Hospital Anxiety and Depression Scale (HADS-A and HADS-D subscales) [25,26]. The dependent variables were quality of life (primary outcome), functional impact of FM, intensity of pain, and mood; the independent variables were sociodemographic and clinical variables [16]. Supplementary material S2 provides a summary of the interpretation of the measurement scales of the dependent variables. Due to the COVID-19 outbreak, between 2020 and 2021, phone interviews and online data collection surveys were implemented in addition to the face-to-face interviews at follow-up.

### 2.4. Statistical Analysis

In this study, the data analyst was blinded to the group allocation. The sample experienced a moderate dropout level (23.7%), in line with expectations for a pragmatic study in PC. For the analysis, all individuals who answered the baseline questionnaires were included, regardless of whether they answered the questionnaires at all follow-ups.

Quantitative variables were summarized as means and standard deviations (SDs), while qualitative variables were reported as frequencies and percentages of patient characteristics for the control and intervention groups, with differences being assessed using Student’s *t* test or the chi-square test, respectively.

The evolution of the outcomes over time was modelled using a mixed linear approach [27]; the five time points were treated as a categorical variable. A random intercept at the subject level was incorporated into the model to accommodate subject baseline variability and the clustered structure of the data. Longitudinal correlation was considered by including an autoregressive first-order residual in the model. To investigate whether the evolution of the outcomes differed by study group, an interaction effect was incorporated into the model. Additionally, evolution was taken into account by including the following variables: age, number of comorbidities (from the following list: muscle disorder, dysmenorrhea, irritable bladder syndrome, myofascial pain, chronic pelvic pain, hyperthyroidism, hypothyroidism), number of triggering factors (composed of prolonged stressful situations, psychological trauma, excess of physical activity, and physical trauma), the number of factors responsible for maintenance, and the number of symptoms (from the following list: fatigue, paresthesia of hands and feet, morning stiffness, urgency to urinate, dryness of mucous membranes, Raynaud’s syndrome, intolerance to olfactory and auditory stimuli, non-specific low back pain, cephalic instability, increased sensitivity to side effects of drugs). Regarding the factors responsible for maintenance, they are physical, psychological, or psychosocial factors (such as a physical or a psychological trauma and excessive workload, respectively) probably responsible for maintaining fibromyalgia symptoms. The final model included only statistically significantly associated factors.

Based on the final model, the marginal mean of the outcome variable was estimated for each study group at each study time. Additionally, the marginal means between the groups were compared at each study time and the confidence intervals for the mean differences were calculated. The overall type-I error rate was set at 5%. Statistical analyses were performed using R (v4.2.2). Reporting was guided by consolidated standards of reporting trials (CONSORT) for parallel group randomized trials [28].

## 3. Results

**Figure 1:**
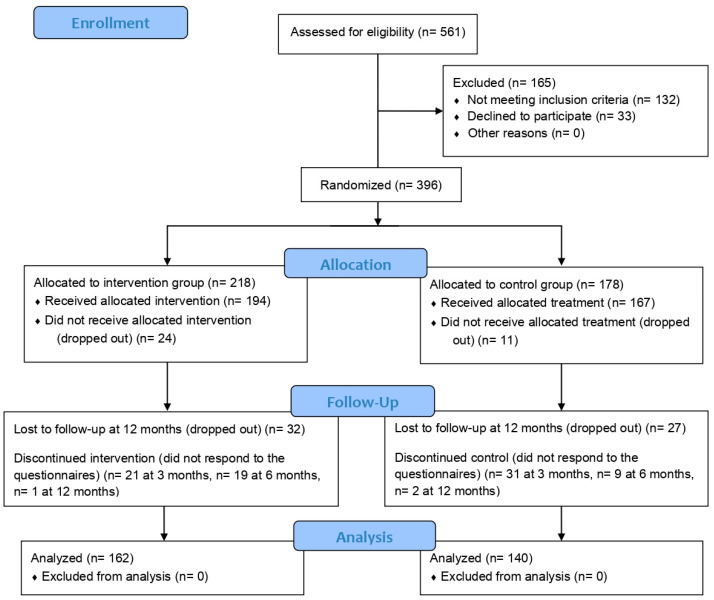
Flow diagram.

The evaluation of the FIBROCARE Program included the results of five waves, collected between April 2017 and January 2020, with follow-up ending in March 2021. A total of 302 patients participated in the program: 140 in the control group and 162 in the intervention group (Figure 1). These numbers exceed the estimated minimum sample size for this study (260 individuals, 130 per study arm) [16].

Overall, 23.7% of the initial randomized sample (n = 396) had missing data (25.7% and 21.3% for the intervention and control groups, respectively). With respect to the data missing at each stage, 31 patients (17.4%) in the control group did not respond to the questionnaires at 3 months follow-up, while 21 patients (9.6%) in the intervention group did not respond. At 6 months, there were fewer losses, involving 9 (5.1%) and 19 (8.7%) individuals in the control and intervention groups, respectively. At 12 months, only 2 (1.1%) and 1 (0.5%) individual(s) in the control and intervention groups, respectively, were lost.

The sample overwhelmingly comprised women (97%), and the mean age of the patients was 61.5 years (SD = 10.7). The mean time since FM diagnosis was 7.05 years (SD = 5.98) and patients had an average of 4.3 symptoms (SD = 2.26). The most frequent comorbidities were chronic pelvic pain (in 42.7% of patients) and muscle disorders (23.2%). Overall, 83.4% of patients had some type of triggering factor, 54.3% with one and 23.8% with two types. The most frequent triggering factor was prolonged stressful situations (in 47.7% of patients), followed by psychological trauma (27.8%), excess physical activity (24.8%), and physical trauma (19%). Of the factors responsible for maintaining FM, physical or psychological factors were the cause in 42.1% of patients, and psychosocial factors were responsible for 25.5% of cases. We also note that 72.8% of the patients had memory impairment, 67.9% had difficulty concentrating, and 35.4% experienced disturbed sleep. A family history of FM was present for 27.5% of the patients. Patients in the intervention group participated in an average of 9.70 (SD = 2.32) sessions (Table 1).

There were no statistically significant differences between the control and intervention groups with respect to the baseline information collected on sex, age, comorbidities, triggering factors, factors responsible for maintenance, the number of symptoms, and years since FM diagnosis.

### 3.1. Evolution of Outcomes over the Study Period

#### 3.1.1. Quality of Life (SF-36)

Patient quality of life was analyzed with respect to the physical (PCS) and mental (MCS) component summary scales and their component dimensions.

##### Physical Component Summary Scale and Dimensions

The PCS score was related to the number of symptoms, study group and time, and the interaction of the latter two variables. At baseline, the mean scores were almost identical in the two groups: 28.32 and 29.94 in the control and intervention groups, respectively (Table 1). The number of symptoms was associated with lower quality of life, measured as the PCS (beta = −0.77, *p* < 0.001) and of all of its dimensions. Additionally, we note that increasing patient age was associated with poorer Physical Functioning (PF) (beta = −0.15, *p* < 0.001) (Table 2).

**Figure 2:**
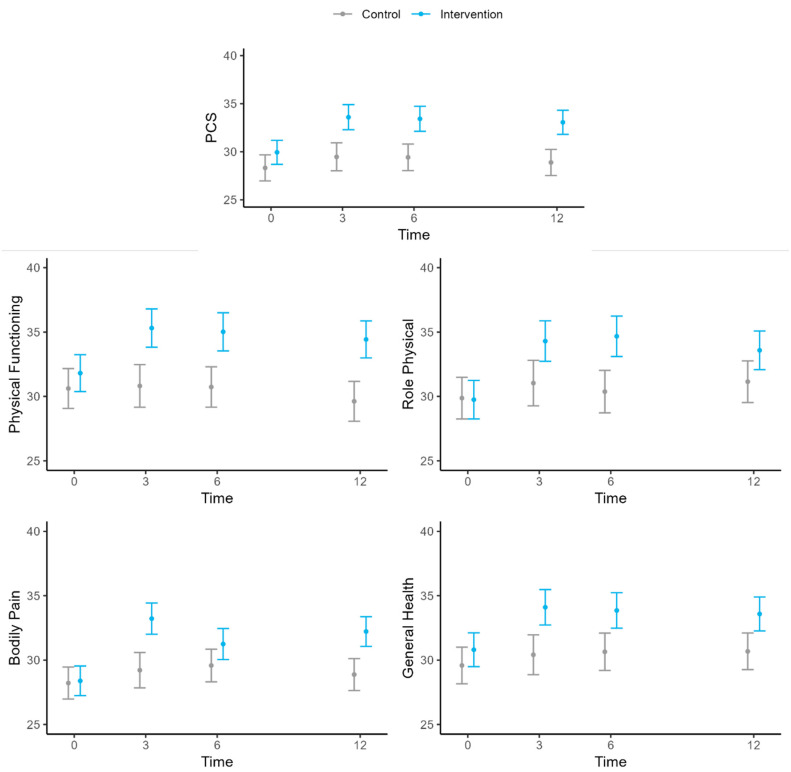
Evolution of SF-36 physical health domains over time. PCS, physical component summary score of the Short-Form 36 (SF-36) Health Survey.

At 3-, 6-, and 12-month follow-ups, the PCS values of the control group had not changed relative to the baseline. However, in the intervention group, an increase was observed at the 3-month follow-up, which was maintained at 6 and 12 months (Table 3, Figure 2).

The mean increase in the PCS of the intervention group relative to the control at 3 months was 4.13 points (95% CI: 6.08, 2.17). At 6 and 12 months, the increases were 4.0 (95% CI: 5.90, 2.10) and 4.17 (95% CI: 6.02, 2.33), respectively. The results for the PF, Role Physical (RP), Bodily Pain (BP), and General Health (GH) dimensions were similar to those obtained for the PCS (Table 3, Figure 2).

##### Mental Component Summary Scale and Dimensions

**Figure 3:**
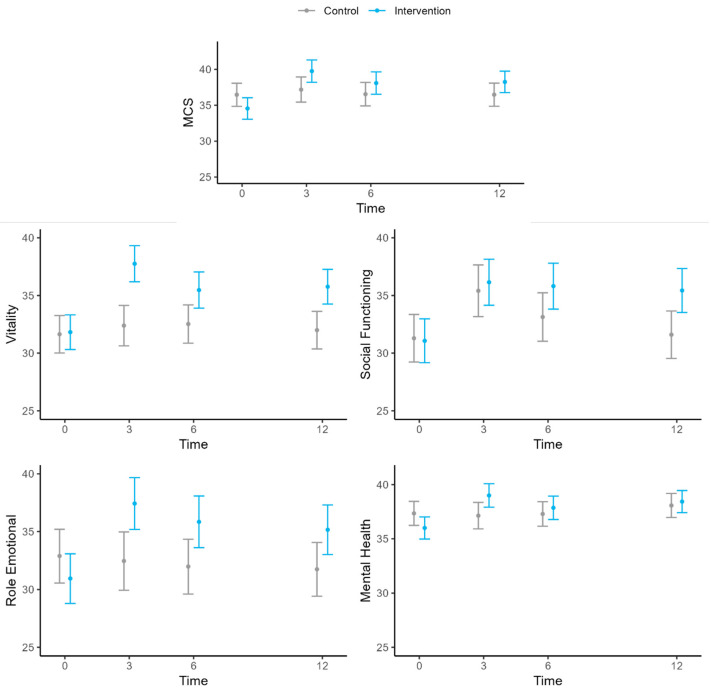
Evolution of SF-36 Mental Health domains over time. MCS, mental component summary score of the Short-Form 36 (SF-36) Health Survey.

The MCS score was related to the number of symptoms, years since FM diagnosis, study group and time, and the interaction of these latter two variables. At baseline, there were no significant differences between groups (Table 1). However, at the 3-month follow-up, the intervention group had a 2.58-point higher mean score than the control group (95% CI: 4.92, 0.23), but this difference was not sustained at 6 and 12 months (Table 3, Figure 3).

In the Social Functioning (SF) dimension, the effect of the intervention was only seen after 12 months of follow-up (3.83; 95% CI: 6.64, 1.02), while an effect was apparent by 3 months for the Mental Health (MH) dimension (1.87; 95% CI: 3.50, 0.23), but it was not sustained in the subsequent follow-ups. On the other hand, the intervention group had higher mean Vitality (VT) and Role Emotional (RE) dimension scores than the control group in all the follow-ups (Table 3, Figure 3).

The increasing number of symptoms was associated with poorer quality of life related to MCS (beta = −0.51 *p* = 0.008), and for all of its dimensions except MH. We also note that the increasing number of years since FM diagnosis was associated with better quality of life, measured as MCS (beta = 0.20, *p* = 0.007) and MH (beta = 0.15, *p* < 0.001) (Table 4).

#### 3.1.2. Impact of Fibromyalgia

**Figure 4:**
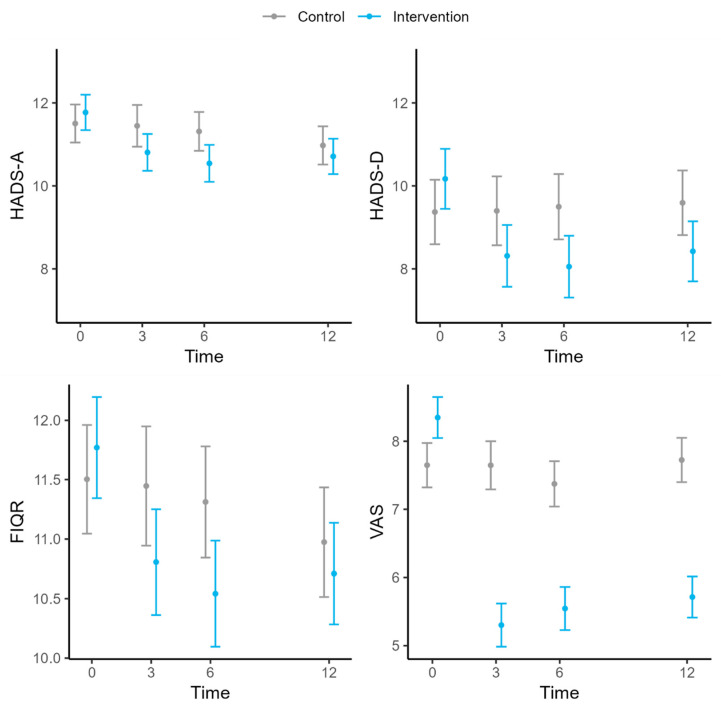
Evolution of HADS-A, HADS-D, FIQR, and VAS scores over time. HADS-A, Hospital Anxiety and Depression Scale—anxiety subscale; HADS-D, Hospital Anxiety and Depression Scale—depression subscale; FIQR, Revised Fibromyalgia Impact Questionnaire; VAS, Visual Analog Scale.

In relation to the impact of FM, measured by the FIQR, it is associated with the number of symptoms, the study group and time, and the interaction of the latter two variables. This model showed that the initial mean FIQR values did not differ significantly between the two groups (Table 1). The mean FIQR of the control group did not change significantly over time, but the intervention group experienced a drop in the mean value at the 3-month follow-up that was maintained at 6 and 12 months. The mean FIQR of the intervention group was 17.68 points lower than that of the control group (95% CI: 13.03, 22.33) at the 3-month follow-up, 12.46 (95% CI: 7.93, 16.99) at 6 months, and 14.9 (95% CI: 10.47, 19.33) at 12 months (Table 3, Figure 4). The number of symptoms was also positively associated with the impact of FM, wherein FIQR scores increased by 1.9 points, on average, for each additional symptom (*p* < 0.001) (Table 5).

#### 3.1.3. Intensity of Pain

The intensity of pain was found to be associated with the study group and time, and their interaction. The intervention group had a higher mean VAS score than the control group at baseline (Table 1). The VAS values of the control group did not change over the study period, but a decrease in VAS score was observed in the intervention group at the 3-month follow-up, which was maintained at 6 and 12 months. After 3 months, the mean VAS score of the intervention group was 2.34 points lower (95% CI: 1.87, 2.82) than for the control group, and at the 6- and 12-month follow-ups, the mean scores were 1.83 (95% CI: 1.37, 2.29) and 2.01 (95% CI: 1.57, 2.46) lower, respectively (Table 3, Figure 4).

#### 3.1.4. Anxiety and Depressive Symptoms

Anxiety and depressive symptoms were related to the number of symptoms, study group, and time, and the interaction of the latter two variables. For anxiety, no differences between the groups in the mean HADS-A subscale score were observed at baseline (Table 1) or after 3 months of follow-up (Table 3, Figure 4). After 6 months, the HADS-A value was significantly lower in the intervention group than in the controls, indicating an improvement in their anxiety symptoms. The average decrease was 0.77 points (95% CI: 0.12, 1.42) but this improvement was not maintained after 12 months of follow-up. The result for depression was similar to that for anxiety, although the improvement observed at the 6-month follow-up was maintained after 12 months (1.17; 95% CI: 0.11, 2.23) (Table 3, Figure 4). We note that the more symptoms that were present, the worse were the symptoms of anxiety (beta = 0.22, *p* < 0.001) and depression (beta = 0.38, *p* < 0.001) (Table 5).

## 4. Discussion

In the present study, we have demonstrated the effectiveness of a 12-week non-pharmacological multicomponent group program for FM patients based on physical exercise, CBT, and health education, compared to the usual clinical care (an individual approach based primarily on pharmacological treatment). The FIBROCARE Program was carried out in the public PC service of Catalonia and was developed by a multidisciplinary team (general practitioner, PC nurse, physiotherapist, psychologist). A standardized program of participatory design with the professionals involved in the program was implemented.

Improvement was achieved in the self-perceived intensity of pain, FM functional impact, and physical health in the MT group compared with the control group at the end of the 12-week group sessions, with an effect lasting for up to 12 months.

Different results were noted for the other outcomes. The improvement in the SF-36 MCS score was not maintained over time, with its effect no longer being apparent after 6 months. Considering the dimensions comprising the MCS, the beneficial effect of the intervention was observed only at 3 months in the case of the MH dimension or was very delayed in the case of the SF dimension, being observed only at 12 months. However, good results were obtained for the VT and RE dimensions for up to 12 months. Regarding the VT dimension, patients from the MT group were less likely than those of the control group to perceive that they were constantly tired and fatigued. In the case of the RE dimension, MT group patients were less likely than control patients to have problems with their work and other daily activities due to emotional problems.

In relation to the HADS subscales, we found that the effect of reducing the anxiety symptoms in the MT group occurred late, at 6 months, but was lost at 12 months. A late reduction in the symptoms of depression was also observed at 6 months, which was maintained at 12 months.

The worst possible score in the SF-36 MH dimension encompasses a response of “the feeling of anguish and depression all the time” [29]. In our study, we interpreted patients’ mood by considering jointly the scores of the SF-36 MH dimension and the HADS subscales. We concluded that the initial improvement in patients’ mood was not maintained over time, with anxiety symptoms persisting, although a reduction in depressive symptoms was observed. This conclusion is supported by the previously published qualitative assessment of this program. The patients rated the program well in general, but expressed the need to be heard and to convey their feelings and concerns in a clear reference to their suboptimal mood state. They demanded regular access to psychological treatment in routine clinical care, continuous psychological support being the most frequently highlighted need [18]. In the future, the program will need to be reinforced to promote better psychological support for patients. New studies are necessary to identify the ideal treatment for patients whose mood does not improve under the current program.

Regarding the SF-36 SF dimension, the worst score corresponds to a patient perceiving “extreme and very frequent interference with normal social activities, due to physical or emotional problems” [29]. Several studies have reported that FM patients have significant difficulties in maintaining a satisfactory social life due to the daily limitations imposed by the syndrome [2,30]. Our multicomponent program did not have the objective of improving social interaction. However, a group effect was observed, as described in our previous qualitative studies [17,18], that could help explain the higher score for SF in the MT group at 12 months. Our qualitative studies indicated how the MT group offered patients an opportunity to create new social networks, helping to legitimize FM through patients’ interactions with others diagnosed with the same disease and who face similar challenges. Some patients in our study reported maintaining contact and holding meetings outside of group hours, indicating an emerging pattern of social interaction. Other studies have reported the benefit of a “group effect” on FM patients [31,32]. Participation in a treatment group as a trigger of social interaction and its association with health outcomes would merit better assessment in FM programs.

Our study demonstrated that the more symptoms that are present, the worse the quality of life, mood, and impact of FM become. These outcomes are to be expected if we consider that, during the course of their disease, an FM patient can present a variety of symptoms that may occur concomitantly [33] and determine differences in the self-perception of their state of health. We also found that the longer the time since FM diagnosis, the better a patient’s mood (assessed as the SF-36 MH) tended to be. This finding may be related to the fact that over the course of the illness, patients go through some progressive phases: onset, progression toward diagnosis, acceptance, and development of an effective management strategy [34]. We hypothesize that with the passage of time, patients learn to self-manage their illness and probably live with it better from an emotional perspective. This result suggests that reinforcing patients’ psychological support in the first years after diagnosis is essential.

Considering the elements of our multicomponent approach, recent evidence has shown that physical activity is the main treatment, and that this proves effective in different modalities: walking (the best method), flexibility, strength, stretching in water and on land, and body–mind exercises [35]. The studies reveal beneficial results for approaches that include mixed physical exercise and, above all, a consideration of patient’s preferences and physical abilities with regard to improving adherence. In terms of psychological therapy, CBT and acceptance and commitment therapy are among the most widely studied approaches. They have proved to be effective in decreasing depression and anxiety in FM [36]. In health education, the promotion of education sessions that include day-to-day topics, such as nutrition, sleep, memory, pharmacological usage, and sexuality, is a way of promoting changes in behavior that lead to better self-care and that could positively influence symptom management [37]. Finally, the introduction of pain neuroscience education (PNE) in multicomponent treatments is a novel innovation in FM health education that is proving effective when combined with exercise therapy for improving functional status, pain-related symptoms, depression, and anxiety [38].

Several multicomponent FM programs have been evaluated in non-randomized trials, prospective or before-and-after studies, but there have been few RCT evaluations [39,40,41,42]. Most of these RCTs have been carried out in the hospital setting and, to our knowledge, only one was in a PC setting [39]. In the latter study [39], a 10-week multidisciplinary pilot intervention with health education and physical exercise was performed and compared with the usual care regime. In the education sessions, aspects related to pain education, cognitive behavioral strategies for stress, nutrition, and peer support were developed. The authors evaluated the impact of FM, depression, and anxiety with the Fibromyalgia Impact Questionnaire (FIQ) and HADS, and found good results for all outcomes just after the intervention. The relief of depression and anxiety was maintained at 3 months, but the impact of FM was not. The results were affected by a loss to follow-up at 3 months that limited the maintenance of some effects. In the hospital setting, Martin et al. [41] adopted an approach with health education, physical therapy, and CBT of 12 sessions over 6 weeks, with a follow-up at 6 months. A significant improvement in functional capacity measured as total FIQ scores was noted. There was a significant improvement in perceived pain intensity at 6 months measured by the FIQ, but not by the VAS. A hospital-based RCT in Spain [40] compared the usual care with an MT with PNE as its main component, and exercises (stretching, balance training, posture correction, and low-impact walking). After a 12-week program, the authors obtained improvements in functional impairment, pain, and fatigue, measured by the FIQR, and anxiety and depression, measured by the HADS. Cedraschi et al. [42] adopted an approach based on self-management, pool exercises, and education, consisting of 12 sessions over 6 weeks, with a follow-up at 6 months. Quality of life, according to some SF-36 scales (GH, PF, RP, and SF) and the Psychological General Well-Being Index (PGWBI), was evaluated. The authors reported significant improvements only in the subscales of anxiety and vitality and in the total score of the PGWBI, and improvements in the fatigue, depression, and total FIQ scores.

The two RCTs featuring the longest follow-ups over time (at 6 months) [41,42] yielded a significant improvement in the impact of FM measured by the FIQ, similar to the result we obtained in our study with the FIQR used for up to 12 months. However, in relation to pain intensity, Martin et al. [41] achieved a reduction in pain in the final week, measured with the FIQ, but not in current pain assessed on the VAS scale. Cedraschi et al. [42] found no benefit, in contrast with our study, in which we found a sustained reduction in pain intensity for up to 12 months, as measured by the VAS scale. We recognize that direct comparisons of our results with other published findings are hampered because the previously published RCTs were very different with respect to their multicomponent content, number of sessions, timing of follow-ups, and measurement of the outcomes.

### Strengths and Limitations

The novelty of the present study is the evaluation of a multicomponent program for FM patients in the PC setting, developed by PC professionals and with a follow-up of up to 12 months. As far as we know, this is the first study to evaluate an FM multicomponent program at 12 months. A program that can be implemented in the PC setting is of fundamental relevance for promoting improved accessibility, reducing waiting lists, and encouraging longitudinality [8,43].

The study was carried out in the public health system within the framework of a program developed by the USCSS from the Terres de l’Ebre region. The USCSSs were created by the Generalitat de Catalunya in 2016 with the aim of improving the diagnostic and therapeutic process of people with central sensitivity syndromes. The fact that our study was performed in a specific context and covered only one region of the Spanish nation may make its results and policy implications difficult to generalize to other populations and communities.

The study has other limitations. The most important of these arose from the huge disruption of the health system during the lockdown imposed by the Spanish government during and after the COVID-19 pandemic, which harmed the progress of the project. Project activities were gradually resumed in the post-confinement period, and, to complete the scheduled follow-ups, the program was adapted to fit a mixed format, as previously described. Despite the changes introduced, the methodological rigor of the follow-ups, in an online format or by telephone, was maintained to guarantee the quality of the completed questionnaires.

Another limitation was the variation in the distribution of FM experts across this Territory of Catalonia. This was a new role created by the project, and the personnel included general practitioners and PC nurses acting as health professionals. They had only a certain number of hours to carry out the project tasks, as described by Caballol Angelats et al. [19]. The number of FM experts varied from one to three across PC centers depending on the resources available locally, and over time. This variation in the number of FM experts may have influenced the leadership and organizational dynamics of groups, but not the standardized content. Regarding baseline data, the variables “education” and “living alone” had different distributions in the control and intervention groups. However, the absence of significant interactions of these variables in the study group (control and intervention) in the multivariate models indicates that the effect of the MT is independent of these two variables. Finally, in this study, both patients and treatment providers were aware of their group assignments, which may have increased the risk of expectation bias; only the data analyst was blinded.

## 5. Conclusions

The FIBROCARE Program, a primary care-based model developed in Catalonia (Spain), was effective at 12 months in reducing FM patients’ self-perceptions of the intensity of pain, functional impact, physical health, fatigue, and emotional problems that affect daily activities when compared with the usual clinical care. However, the program was not effective in changing the mood state, since anxiety symptoms tended to persist. The program needs to be reinforced through psychological support, with a special focus on the first years after diagnosis, when the patient’s mood is generally lower. The benefit of the group effect that enhances patients’ socialization is intriguing and suggests that more research is needed in this area.

## Figures and Tables

**Table 1:** Sociodemographic profile of participants at baseline.

	All Patients	Control	Intervention	*p* Value
	N = 302	N = 140	N = 162	
Sex (woman), n (%)	293 (97.0)	135 (96.4)	158 (97.5)	0.738
Age, mean (SD)	61.5 (10.7)	62.6 (11.0)	60.6 (10.3)	0.105
Civil status, n (%)				0.138
Married	211 (76.2)	92 (70.8)	119 (81.0)	
Divorced	36 (13.0)	19 (14.6)	17 (11.6)	
Single	13 (4.69)	7 (5.38)	6 (4.08)	
Widowed	17 (6.14)	12 (9.23)	5 (3.40)	
Education, n (%)				0.003
None	54 (19.6)	17 (13.1)	37 (25.3)	
Primary	138 (50.0)	67 (51.5)	71 (48.6)	
Secondary	64 (23.2)	30 (23.1)	34 (23.3)	
Higher/university	20 (7.25)	16 (12.3)	4 (2.74)	
Employment status, n (%)				0.252
Employed (active)	106 (38.3)	47 (36.2)	59 (40.1)	
Unemployed	29 (10.5)	16 (12.3)	13 (8.84)	
Retired	71 (25.6)	39 (30.0)	32 (21.8)	
Disabled	25 (9.03)	8 (6.15)	17 (11.6)	
Homemaker	46 (16.6)	20 (15.4)	26 (17.7)	
Living alone, n (%)	25 (8.28)	19 (13.6)	6 (3.70)	0.004
Years since FM diagnosis, mean (SD)	7.05 (5.98)	7.33 (5.79)	6.80 (6.14)	0.445
No. of symptoms (SD)	4.30 (2.26)	4.42 (2.34)	4.20 (2.19)	0.407
No. of comorbidities, mean (SD)	1.27 (1.20)	1.36 (1.22)	1.20 (1.17)	0.269
No. of triggering factors, mean (SD)	1.19 (0.80)	1.23 (0.70)	1.16 (0.88)	0.457
No. of factors responsible for maintenance, mean (SD)	1.10 (0.78)	1.16 (0.74)	1.04 (0.81)	0.154
No. of sessions, mean (SD)	-	-	9.70 (2.32)	-
SF-36 PCS, mean (SD)	29.17 (7.79)	28.32 (7.22)	29.94 (8.19)	0.056
SF-36 MCS, mean (SD)	35.44 (10.07)	36.47(10.31)	34.50(9.81)	0.103
FIQR, mean (SD)	66.20 (18.6)	67.36 (17.56)	67.27 (19.55)	0.328
VAS, mean (SD)	8.03 (1.79)	7.65 (1.78)	8.35 (1.74)	0.001
HADS-A, mean (SD)	11.6 (2.93)	11.5 (2.95)	11.7 (2.91)	0.535
HADS-D, mean (SD)	9.79 (4.54)	9.41 (4.53)	10.1 (4.53)	0.180

N, total number of cases; n, number of cases; %, percentage of cases; SD, standard deviation; SF-36 PCS, physical component summary score of the Short-Form 36 (SF-36) Health Survey; SF-36 MCS, mental component summary score of the SF-36 Health Survey; FIQR, Revised Fibromyalgia Impact Questionnaire; VAS, Visual Analog Scale; HADS-A, Hospital Anxiety and Depression Scale—anxiety subscale; HADS-D, Hospital Anxiety and Depression Scale—depression subscale.

**Table 2:** Mixed linear model for the physical component summary score of the SF-36 Health Survey and scoring of its subscales.

		PCS	Physical Functioning	Role Physical	Bodily Pain	General Health
	Variable	Estimate (SE)	Estimate (SE)	Estimate (SE)	Estimate (SE)	Estimate (SE)
	Intercept	31.62 (1.0) *	42.93 (2.89) *	33.07 (1.16) *	30.45 (0.87) *	32.63 (1.03) *
Group	Control	1	1	1	1	1
	Intervention	1.62 (0.94)	1.19 (1.07)	−0.12 (1.12)	0.17 (0.86)	1.22 (0.98)
Time	Basal	1	1	1	1	1
	3 mo	1.15 (0.70)	0.19 (0.79)	1.17 (0.96)	0.99 (0.79)	0.83 (0.83)
	6 mo	1.1 (0.71)	0.11 (0.75)	0.50 (0.91)	1.36 (0.75)	1.06 (0.78)
	12 mo	0.57 (0.71)	−0.99 (0.74)	1.28 (0.90)	0.66 (0.73)	1.09 (0.77)
No. symptoms		−0.77 (0.16) *	−0.74 (0.19) *	−0.74 (0.19) *	−0.52 (0.14) *	−0.71 (0.17) *
Age (years)		-	−0.15 (0.04)*	-	-	-
Interaction	Intervention: 3 mo	2.50 (0.94) *	3.30(1.06) *	3.38 (1.29) *	3.84 (1.06) *	2.47 (1.11) *
	Intervention: 6 mo	2.38 (0.98) *	3.10 (1.03) *	4.42 (1.25) *	1.50 (1.02)	1.99 (1.07)
	Intervention: 12 mo	2.55 (0.97) *	3.62 (1.00) *	2.55 (1.22) *	3.17 (0.99) *	1.68 (1.05) *
SD random intercept		5.54	6.94	6.19	4.27	5.63
SD residual		5.91	6.09	7.42	6.08	6.37
Autocorrelation		0.55	-	-	-	-

PCS, physical component summary score of the Short-Form 36 (SF-36) Health Survey; SD, standard deviation; SE, standard error; mo, months; *, statistically significant coefficient.

**Table 3:** Mean differences between control and intervention groups by study time.

	Baseline	3 Months	6 Months	12 Months
SF-36 PCS	−1.62 (−3.47, 0.23)	−4.13 (−6.08, −2.17) *	−4.00 (−5.90, −2.10) *	−4.17 (−6.02,−2.33) *
Physical Functioning	−1.19 (−3.30, 0.92)	−4.49 (−6.72, −2.27) *	−4.29 (−6.45, −2.13) *	−4.81 (−6.92, −2.69) *
Role Physical	0.12 (−2.08, 2.33)	−3.26 (−5.63, −0.89) *	−4.30 (−6.58, −2.02) *	−2.43 (−4.64, −0.23) *
Bodily Pain	−0.17 (−1.86, 1.52)	−4.01 (−5.84, −2.17) *	−1.67 (−3.42, 0.09)	−3.34 (−5.04, −1.65) *
General Health	−1.22 (−3.16, 0.72)	−3.69 (−5.77, −1.62) *	−3.21 (−5.21, −1.21) *	−2.90 (−4.84, −0.96) *
SF-36 MCS	1.91 (−0.28, 4.11)	−2.58 (−4.92, −0.23) *	−1.55 (−3.81, 0.71)	−1.78 (−3.98, 0.42)
Vitality	−0.18 (−2.40, 2.03)	−5.37(−7.71, −3.02) *	−2.94 (−5.22, −0.66) *	−3.77 (−5.98, −1.55) *
Social Functioning	0.22 (−2.59, 3.03)	−0.74 (−3.74, 2.26)	−2.67 (−5.57, 0.22)	−3.83 (−6.64, −1.02) *
Role Emotional	1.95 (−1.21, 5.11)	−4.98 (−8.35, −1.60) *	−3.87 (−7.12, −0.62) *	−3.42 (−6.58, −0.26) *
Mental Health	1.35 (−0.16, 2.85)	−1.87 (−3.50, −0.23) *	−0.57 (−2.14, 0.99)	−0.36 (−1.87, 1.15)
FIQR	1.68 (−2.74, 6.10)	17.68 (13.03, 22.33) *	12.46 (7.93, 16.99) *	14.9 (10.47,19.33) *
VAS	−0.70 (−1.15; −0.26) *	2.34 (1.87; 2.82) *	1.83 (1.37; 2.29) *	2.01 (1.57; 2.46) *
HADS-A	−0.27 (−0.89, 0.36)	0.64 (−0.03, 1.31)	0.77 (0.12, 1.42) *	0.26 (−0.36, 0.89)
HADS-D	−0.80 (−1.86, 0.26)	1.09 (−0.03, 2.20)	1.44 (0.36, 2.53) *	1.17 (0.11, 2.23) *

SF-36 PCS, physical component summary score of the Short-Form 36 (SF-36) Health Survey; SF-36 MCS, mental component summary score of the SF-36 Health Survey; FIQR, Revised Fibromyalgia Impact Questionnaire; VAS, Visual Analog Scale; HADS-A, Hospital Anxiety and Depression Scale—anxiety subscale; HADS-D, Hospital Anxiety and Depression Scale—depression subscale; *, statistically significant mean differences (*p* < 0.05).

**Table 4:** Mixed linear model for the mental component summary score of the SF-36 Health Survey and scoring of its subscales.

		MCS	Vitality	Social Functioning	Role Emotional	Mental Health
	Variable	Estimate (SE)	Estimate (SE)	Estimate (SE)	Estimate (SE)	Estimate (SE)
	Intercept	37.28 (1.29) *	36.1 (1.18) *	35.28 (1.51) *	35.42 (1.69) *	36.28 (0.65) *
Group	Control	1	1	1	1	1
	Intervention	−1.91 (1.12)	0.18 (1.1)	−0.22 (1.43)	−1.95 (1.60)	−1.35 (0.76)
Time	Basal	1	1	1	1	1
	3 mo	0.72 (0.92)	0.75 (0.86)	4.11 (1.17) *	−0.43 (1.33)	−0.21 (0.72)
	6 mo	0.08 (0.87)	0.89 (0.91)	1.84 (1.11)	−0.91 (1.25)	−0.06 (0.68)
	12 mo	0.01 (0.85)	0.36 (0.93)	0.31 (1.09)	−1.15 (1.23)	0.73 (0.67)
No. symptoms		−0.51 (0.19) *	−0.51 (0.19) *	−0.93 (0.25) *	−0.59 (0.27) *	-
Years since FM diagnosis		0.20 (0.07) *	-	-	-	0.15 (0.04) *
Interaction	Intervention: 3 mo	4.49 (1.23) *	5.18 (1.15) *	0.96 (1.57)	6.93 (1.78) *	3.21 (0.97) *
	Intervention: 6 mo	3.46 (1.19) *	2.76 (1.2) *	2.90 (1.52)	5.82 (1.72) *	1.92 (0.94) *
	Intervention: 12 mo	3.69 (1.16) *	3.58 (1.27) *	4.05 (1.48) *	5.37 (1.68) *	1.71 (0.92)
SD random intercept		6.54	5.85	8.36	9.36	3.52
SD residual		7.07	7.75	9.04	10.21	5.59
Autocorrelation		-	0.66	-	-	-

MCS, mental component summary score of the Short-Form 36 (SF-36) Health Survey; SD, standard deviation; SE, standard error; mo, months; *, statistically significant coefficient.

**Table 5:** Mixed linear model for FIQR, VAS, HADS-A, and HADS-D scores.

		FIQR	VAS	HADS-A	HADS-D
	Variable	Estimate (SE)	Estimate (SE)	Estimate (SE)	Estimate (SE)
	Intercept	58.84 (2.42) *	7.65 (0.17) *	10.57 (0.33) *	7.75 (0.60) *
Group	Control	1	1	1	1
	Intervention	−1.68 (2.24)	0.70 (0.22) *	0.27 (0.32)	0.80 (0.54)
Time	Basal	1	1	1	1
	3 mo	−3.47 (1.60) *	−0.002 (0.19)	−0.06 (0.27)	0.03 (0.38)
	6 mo	−2.50 (1.64)	−0.28 (0.20)	−0.19 (0.25)	0.13 (0.35)
	12 mo	0.60 (1.64)	0.08 (0.21)	−0.53 (0.25) *	0.22 (0.35)
No. symptoms		1.93 (0.40) *	-	0.22 (0.05) *	0.38 (0.10) *
Interaction	Intervention: 3 mo	−1.60 (2.15) *	−3.05 (0.25) *	−0.90 (0.36) *	−1.88 (0.50) *
	Intervention: 6 mo	−10.78 (2.26) *	−2.53 (0.28) *	−1.04 (0.35) *	−2.24 (0.49) *
	Intervention: 12 mo	−13.22 (2.25) *	−2.71 (0.28) *	−0.53 (0.34)	−1.97 (0.47) *
SD random intercept		13.8	0.86	1.77	3.66
SD residual		13.7	1.75	2.09	2.89
Autocorrelation		0.58	0.69	-	-

SD, standard deviation; SE, standard error; mo, months; *, statistically significant coefficient; FIQR, Revised Fibromyalgia Impact Questionnaire; VAS, Visual Analog Scale; HADS-A, Hospital Anxiety and Depression Scale—anxiety subscale; HADS-D, Hospital Anxiety and Depression Scale—depression subscale.

## Data Availability

The datasets used and analyzed during this study are available from the corresponding author upon reasonable request. Data sharing will have to follow appropriate regulations.

## Supplementary Materials

The following supporting information can be downloaded at: https://www.mdpi.com/article/10.3390/jcm14010161/s1, Supplementary material S1: The FIBROCARE Program [9,13,15,16,19,35,36,38,44,45,46,47,48,49,50,51]; Supplementary material S2: Dependent variables, measurement instruments, and interpretation of the scales [20,21,22,23,24,25,26,29,52].

## Abbreviations

BP: Bodily Pain dimension of the SF-36; CBT: cognitive–behavioral therapy; FIQR: Revised Fibromyalgia Impact Questionnaire; FIQ: Fibromyalgia Impact Questionnaire; FM: fibromyalgia; GH: General Health dimension of the SF-36; HADS: Hospital Anxiety and Depression Scale; HADS-A: Hospital Anxiety and Depression Scale—anxiety subscale; HADS-D: Hospital Anxiety and Depression Scale—depression subscale; ICS: Institut Català de la Salut; MCS: mental component summary score of the SF-36; MH: Mental Health dimension of the SF-36; MT: non-pharmacological multicomponent group therapy; PC: primary health care; PCCs: primary care centers; PCS: physical component summary score of the SF-36; PF: Physical Functioning dimension of the SF-36; PNE: pain neuroscience education; RCT: randomized controlled trials; RE: Role Emotional dimension of the SF-36; RP: Role Physical dimension of the SF-36; SF: Social Functioning dimension of the SF-36; SF-36: Short-Form 36 v2 Health Survey questionnaire; USCSS: Unit Specialized in Central Sensitivity Syndromes; VAS: Visual Analog Scale; VT: Vitality dimension of the SF-36.
