# Supplementary material for: Effectiveness of a Multicomponent Program for Fibromyalgia Patients in a Primary Care Setting (FIBROCARE Program): A Pragmatic Randomized Controlled Trial
Source: J Clin Med. 2024 Dec 30;14(1):161. doi: 10.3390/jcm14010161 (PMC11721895; doi:10.3390/jcm14010161)
Supplement: Supplementary file 1 [file jcm-14-00161-s001.zip › jcm-3248671-supplementary.pdf]

Supplementary material S1. The FIBROCARE Program

1. Program objectives

The program adopts a multicomponent and multidisciplinary group approach for patients diagnosed with fibromyalgia (FM), carried out in a primary care (PC) setting. The program is based on health education, therapeutic physical exercise, and cognitive–behavioral therapy (CBT), provided by nurses and general practitioners (GPs), psychologists, and physiotherapists from PC, who provide the intervention in a coordinated manner. The educational strategy of the neuroscience of pain is incorporated.

The program's general objectives have two aspects, one for professionals and the other for patients. For professionals, tools were provided to facilitate and standardize the group and individual approaches by producing content, defining progress indicators, and clarifying the role of each professional involved in the intervention. For patients, the goals were to improve their quality of life, pain, function, mood, and sleep, and to promote a change in lifestyle through the multicomponent therapy, in which they acquire resources to cope with pain in everyday life.

2. Training of FM experts

The training of professionals as "FM experts" was essential to deliver a high-quality group approach. The objectives of the training were to instill knowledge about Central Sensitization Syndromes and, specifically, about FM, to provide the skills to lead groups, to know how to conduct the tests used to monitor the evolution of patients, and to have the expertise to apply the standardized group content covering health education, therapeutic physical exercise, and behavioral activation. The training was aimed at the professionals who had to lead the groups: nurses and referring GPs from each primary care team (PCT).

The professionals who provided the training were from the Unit Specialized in Central Sensitivity Syndromes (USCSS) in Terres de l'Ebre (Tortosa, Catalonia): a coordinating GP, a nurse manager, a psychologist, a physiotherapist, and several expert professionals.

The training was carried out in different phases between 2011 and 2019. First, the core team of a nurse and a GP from the USCSS was set up. The physiotherapist and psychologist were then trained by taking specific courses. The USCSS team trained the FM experts (nurses and GPs from each PC area in the territory). The training was carried out annually and took the form of a 20-hour course and a 10-hour workshop. Twenty FM experts (eleven nurses and nine GPs) were trained. For more details about this training, see Caballol Angelats et al. 2023 [19].

3. Program design

The preparation of the material began two years before the start of the program. The study's design process involved the participation of the USCSS professionals and of the first professionals who were trained as FM experts. After reviewing the literature and guidelines, we concluded that an evidence-based approach was needed, consisting of three components—health education, physical exercise and CBT— and that this approach would ideally be carried out over 12 weeks.

In accordance with the revised bibliography and the clinical characteristics of FM, some evolution indicators were defined with respect to the use of specific tests: quality of life, measured with the Short-Form 36 (SF-36) v2 Health Survey Questionnaire; mood, assessed with the Hospital Anxiety and Depression Scale (HADS-A and HADS-D subscales); pain intensity, measured with the Visual Analog Scale (VAS); and functional impact, assessed by the Revised Fibromyalgia Impact Questionnaire (FIQR).

The design of the program was based on the scientific evidence published up to 2017.

References: [13,15,44]

The following support materials were developed within the framework of the FIBROCARE Program:

a) A computer application designed by the IT team of the Information Systems of the Territorial Management of the Institut Català de la Salut (ICS) in Terres de l'Ebre that can be accessed through the intranet of the ICS, Terres de l'Ebre.

b) An information leaflet for the patient.

c) A syllabus for the sessions in a presentation format with all the health education topics developed, self-completed exercise tables, and a behavioral activation syllabus.

4. Professionals involved in the program

The commitment of each professional involved in the group intervention was agreed upon with the management. Their usual work responsibilities were fulfilled by other professionals in the workplace. The number of working days allocated was two for the motivational interviews before joining the group and half of one or one for each group session (12 weeks running). Each FM expert ran one or two groups a year, making up a total commitment of 28 days.

The role of each professional in the program was defined. The FM experts (nurse and GP) of each PC team recruited the patients by telephone and organized the initial interviews. The group was led by the nurse (FM expert) for the 12 sessions and those in which the tests were done.

Role of each professional by work group

a) USCSS team

Rheumatologist: Confirm the diagnosis of FM and determine the approach to complex cases. The diagnosis of all patients is confirmed by this professional.

General practitioner (GP): Coordinate the USCSS professionals, define the objectives, cultivate a relationship with managers, promote professional training, and review derived cases. Provide support for territorial GP.

Nurse, physiotherapist, and psychologist: Support the FM experts in organizing the groups in each PC team, conduct the sessions, register the data, resolve difficulties, and ensure that the multicomponent group is carried out in a standardized way.

b) FM expert network

Role of the FM expert network: Organize and carry out the group tasks in their PC team, participate in training, and work in coordination with the USCSS team and the professionals involved in the group approach. Liaison with and provide support for the professionals of their PC team.

GP: Participate in the neuroscience-based pain session and provide support for the registry of the tests administered to each patient.

Nurse: Carry out the group and individual treatment plan in coordination with the patient's nurse.

Physiotherapist: Teach the various types of exercises to patients, progressively and in a manner adapted to the circumstances of each patient. Support the nurse who conducted the group in repeating the exercises in the sessions.

Psychologist: Carry out activities in the group related to behavioral activation and foster motivation for change. Support the nurse in imparting relaxation techniques.

c) Primary care team

GP: Manage diagnostic suspicion, differential diagnosis, first approach, and follow-up after the multicomponent therapy in the group; provide pharmacological therapy, if indicated.

Nurse: Carry out the individual care plan and evaluate the proposal to include patients in multicomponent therapy in the group.

5. Components of the group program

a) Health education

The general objective of the health education component is to train the person so that they have sufficient information and knowledge of the disease they suffer from and help them acquire the necessary skills to maintain and/or improve their health, or to be independent (Gencat 2024). Health education is a planned teaching–learning process that leads to the modification of health-related behaviors, by adopting healthy behaviors or changing health-harming behaviors to health-promoting behaviors.

Research has shown that when patients acquire self-management skills, it can lead to significant improvements, particularly in pain reduction. Self-care can improve patients' motivation with respect to their eating routine and exercise, as well as in relation to their

symptoms, especially the improvement of pain. In addition to reducing pain, there is evidence of improved short- and long-term physical function (Geraghty et al. 2021).

In recent years, the inclusion of pain neuroscience education as part of the therapeutic approach, in conjunction with other therapies, has yielded benefits for FM patients, in terms of their quality of life and pain improvement. Pain neuroscience education helps empower the patient by providing knowledge about their pain, motivating them to improve their adherence to the proposed treatment and to acquire more resources to choose coping pathways (Saracoglu et al. 2022). This is incorporated explicitly or implicitly in all the sessions held as part of the group approach, as the basis that supports and gives meaning to the combination and interrelationship of health education, therapeutic exercise, and psychological therapies worked on during the group sessions.

Studies by Gómez-de-Regil (2021) highlight that health education improves various clinical aspects for fibromyalgia (FM) patients, including pain intensity, fatigue, sleep quality, depression, anxiety, cognitive function, and overall quality of life, with pronounced effects on pain and depression. Furthermore, Gálvez-Sánchez et al. (2023) demonstrated benefits across both emotional and physical functions. Importantly, health education is more effective when combined with physical exercise (Kundakci et al. 2022) and is especially impactful as part of a multidisciplinary therapy approach (Kundakci et al. 2022; Gálvez-Sánchez et al. 2023).

References: [9, 38, 45-48]

b) Physical exercise

The beneficial effects of therapeutic exercise on FM have been documented in several studies (Zhang et al. 2022, Sosa-Reina et al. 2017, Bidonde et al. 2019). The main objectives of therapeutic exercise include reduction of pain, improvement of disability and physical function, activation of neuroplasticity mechanisms, and enhanced mood and sleep quality.

These effects are produced by performing a combination of types of exercise, especially multimodal active therapies (combination of land and water aerobic exercise, exergames, mind–body). Passive therapies, such as manual massage and acupuncture, have also shown positive effects on reducing pain, fatigue, and depression, but their effect is short-term. On the other hand, it is very important to know the patient's preferences so that their physical exercise plan can be adapted in order to increase their adherence to the plan.

In the FIBROCARE Program, the four sessions led by the physiotherapist (F) consist of the following:

F1: Learning and practicing stretching exercises;

F2: Learning and practicing joint exercises;

F3: Learning and practicing strength exercises;

F4: Learning and practicing coordination exercises.

References: [35,49,50]

c) Psychological component

Psychological therapies play a key role in the treatment of FM patients. The majority of published studies investigate CBT, which has shown clear benefits. Additionally, mindfulness and other new-generation therapies have demonstrated potential for improvement as well (Pei et al. 2021, Cojocaru et al. 2024).

The main goals of psychological therapy for patients with FM include enhancing quality of life, promoting the reduction of pain, disability, fatigue, anxiety, and depression, and improving sleep.

In the FIBROCARE Program, the behavioral activation is carried out at the group level in small groups of patients. Four consecutive behavioral activation sessions are carried out with the psychologist (P1). The topics of the sessions are as follows:

P1: Activities and mood.

P2: Planning pleasant activities.

P3: Management of difficulties.

P4: Identification of objectives.

References: [36,51]

6. Group sessions of the FIBROCARE Program

The group program consists of one 2-hour session per week for 12 weeks, in groups of 10-12 people, carried out in PC centers. The sessions are always led by two professionals, a group leader and an assistant, one of whom is always the nurse (FM expert). The sessions are timetabled according to the published protocol (Caballol Angelats et al. 2019).

Structure of the sessions

Each session is strictly structured and timed to include, first, a review of how the previous week has gone, followed by a review of tasks (15 min), delivery of the health education syllabus (30 min), and physical exercise (45 min). The session ends with relaxation (20 min) and a presentation of suggested homework (10 min). When provided, psychological therapy replaces the health education component (45 min). To keep to time, the physical exercise component is shortened to 30 min.

With the aim of optimizing the content of the 12 group sessions, the initial protocol was slightly modified by the addition of two more sessions (non-therapeutic), at the beginning and the end of the program, to introduce (or finalize) the group and to perform the evolution tests.

Initial session (non-therapeutic): Introducing the group. Presentation of professionals and group members, and exploration of patients' expectations. Setting out the group's objectives. Explanation of group rules. Group agreements. Group therapeutic engagement. Initial assessment with baseline tests: SF-36v2 Health Survey, VAS, HADS, and FIQR. Professionals involved: nurse (FM expert), physiotherapist, USCSS team.

Session 1. **Introduction to multicomponent therapy**. What is fibromyalgia?; importance of the environment. Inform patients about the general terms of the disease, the methodology of the group intervention, and its content. Physical exercise: Information and completion of the 6-minute walk test. CBT: Strategies based on cognitive–behavioral therapy are explained. Professionals involved: nurse (FM expert), physiotherapist, psychologist, and nurse from the USCSS.

Session 2. **Pain and drugs.** Information about theories of pain. Introduction to the neuroscience of pain. Provision of information about the main medicines advised in FM. Physical exercise: Learning a breathing technique. Relaxation. Professionals involved: nurse (FM expert) and USCSS general practitioner.

Session 3. **Management of attention**. Understand the nature of attention and its role in the perception of pain. Assess the management of attention in everyday life. Practice attention management techniques. Abdominal breathing. Relaxation. Professionals involved: nurse (FM expert) and USCSS general practitioner or USCSS nurse.

Session 4. **Techniques of postural hygiene**. Why do physical exercise? **Physical exercise: stretching (F1)**. Practice exercises. Professionals involved: nurse (FM expert) and physiotherapist.

Session 5. **Nutrition**. Know about the Mediterranean diet—its characteristics and benefits. Chronobiology. Physical exercise: Breathing and review of previous exercises and practice. Relaxation. Professionals involved: nurse (FM expert) and USCSS nurse.

Session 6. **Management of insomnia**. Activities of daily life (ADL). Pain is explained in the context of daily life and guidelines are given to increase work capacity; normalize the pattern of activity; and reduce disability. Guidelines are also provided about sleep: changes in its rhythm and how to deal with them; how to sleep well and wake up better. **Physical exercise: joint exercises and games (F2)**. Relaxation. Professionals involved: nurse (FM expert) and physiotherapist.

Session 7. **Memory**. Concept, external aids, and exercises to preserve memory. Give guidance on how to deal with memory loss and help to preserve memory. Physical exercise: Stretching and joint exercises. Relaxation. Professionals involved: nurse (FM expert) and USCSS nurse.

Session 8. **Sexuality.** Physical exercise: postural hygiene and stretching. Relaxation. Professionals involved: nurse (FM expert) and USCSS nurse.

Session 9. **Activities and mood (P1).** Differentiate mood and behavior. Understand the relationship between doing activities and mood. Types of activities to consider: social interactions, activities that make us feel competent, and activities incompatible with feeling bad or depressed. **Physical exercise: strength (F3) and joint exercises.** Relaxation. Professionals involved: physiotherapist and psychologist.

Session 10. **Planning pleasant activities (P2)**. Know what activities are pleasurable. Scheduling pleasant activities, monitoring conditions such as external pressures, choice of activities that are not highly pleasant, or those that cause excessive anxiety that can interfere with satisfaction or activity. Description of the characteristics of pleasant activities. List of enjoyable activities. Steps to achieve more enjoyable activities. **Physical exercise: coordination (F4).** Relaxation. Professionals involved: physiotherapist and psychologist.

Session 11. **Management of difficulties (P3)**. Identify the obstacles that prevent participation in activities. Solve problems and overcome obstacles. Create a balance in life between stress and fun. Breathing and relaxation. Professionals involved: nurse (FM expert) and psychologist.

Session 12. **Identification of objectives (P4)**. Learn to set realistic goals. Establish short- and long-term goals. Physical exercise: completion of the 6-minute walk test. Relaxation. Professionals involved: physiotherapist and psychologist.

Final session (non-therapeutic): Final assessment of patients and professionals. Summary. Folder of diaries, exercises, and tasks to be given to each patient. Follow-up tests: SF-36v2 Heath Survey, VAS, HADS, and FIQR. Satisfaction survey. Farewell to the group. Professionals involved: nurse (FM expert), psychologist, and physiotherapist.

References: [16]

Supplementary material S2. Dependent variables, measurement instruments, and interpretation of the scales

Dependent variables (measurement instrument)	Summary score or dimension (definition)	Likert scales and interpretation	
		0 (worst score)	100 (better score)
Quality of life (1)	Physical component summary score (physical health and its impact on daily functioning, including physical limitations, pain, and overall health perception)	Significant physical health impairment, with severe limitations or substantial pain	Excellent physical health, with no or minimal physical limitations
	Physical Functioning (ability to perform physical activities)	Very limited in carrying out all physical activities, including bathing or showering, due to health	Performs all types of physical activities, including the most vigorous ones, without any limitations due to health
	Role Physical (limitations in work or activities due to physical problems)	Problems with work or other daily activities due to physical health	No problems with work or other daily activities due to physical health
	Bodily Pain (intensity of pain and its impact on daily life)	Very intense and extremely limiting pain	No pain or limitations due to it
	General Health (overall perception of health)	Rates their own health as poor and believes it may worsen	Rates their own health as excellent
	Mental component summary score (perceived mental and psychological well-being, assessing aspects such as vitality, emotional health, and the social impact of mental health)	Significantly impaired mental health, possibly indicating severe emotional issues	Excellent mental health, with few or no emotional limitations
	Vitality (energy level and fatigue)	Feels tired and exhausted all the time	Feels very energetic and full of energy all the time
	Social Functioning (impact of health on social activities)	Extreme and very frequent interference with normal social activities due to physical or emotional problems	Engages in normal social activities without any interference due to physical or emotional problems
	Role Emotional (limitations in work or activities due to emotional problems)	Problems with work and other daily activities due to emotional problems	No problems with work and other daily activities due to emotional problems
	Mental Health (assessment of psychological well-being, including anxiety, depression, and happiness)	Feelings of distress and depression all the time	Feelings of happiness, tranquility, and calm all the time
Functional impact (2)		0 (low impact of FM)	100 (severe impact of FM)
Intensity of pain (3)		0 (absence of pain)	10 (worst possible pain)
Mood (4)	HADS-A (anxiety)	0 (no anxiety)	21 (severe anxiety)
	HADS-D (depression)	0 (no depression)	21 (severe depression)

(1) Short-Form 36 (SF-36) Health Survey questionnaire; score: 36 items; 8 dimensions, (0-100/dimension)
(2) Revised Fibromyalgia Impact Questionnaire (FIQR); score: 21 items; FIQR total (0-100)
(3) Visual Analog Scale (VAS); score: 0-10
(4) Hospital Anxiety and Depression Scale (HADS); score: 14 items; 2 dimensions, (0-21/dimension)

FM, fibromyalgia

References: [20-26,29,52]
